# The Potential of Proton Therapy for Locally Advanced Breast Cancer: Clinical and Technical Considerations

**DOI:** 10.3390/curroncol30030219

**Published:** 2023-02-28

**Authors:** N. Lalani, S. Alqarni, R. B. Jimenez

**Affiliations:** 1 Department of Radiation Oncology, The Irving Greenberg Family Cancer Centre, Ottawa, ON K2H 8P4, Canada; 2 Department of Radiation Oncology, The Massachusetts General Hospital, Boston, MA 02114, USA

**Keywords:** breast cancer, proton therapy, radiotherapy

## Abstract

Proton therapy is a promising therapeutic modality with unique physical properties that allow for abrupt dose fall-off distal to the target of interest, thereby sparing nearby organs at risk. A number of studies have identified the utility of proton radiation in mitigating treatment related sequelae for patients with locally advanced breast cancers. Thus, in the following review, we highlight clinical and technical considerations for proton radiotherapy delivery in patients with locally advanced breast cancer.

## 1. Background

Oncologic outcomes have improved globally due to major strides across the range of surgery, systemic therapy, radiotherapy and immunotherapy. Radiotherapy continues to be a fundamental part of cancer care, with nearly 50% of patients undergoing radiation at some point in their cancer journey [1]. Traditional radiotherapy approaches utilize X-ray beams, directing photons to the target site. The precision of photon radiation has improved considerably over recent decades due to various technological advances including intensity modulated radiation therapy (IMRT) and stereotactic and image guided radiation therapy. However, unintentional dose deposition to surrounding organs continues to be a treatment-limiting factor.

**Figure 1:**
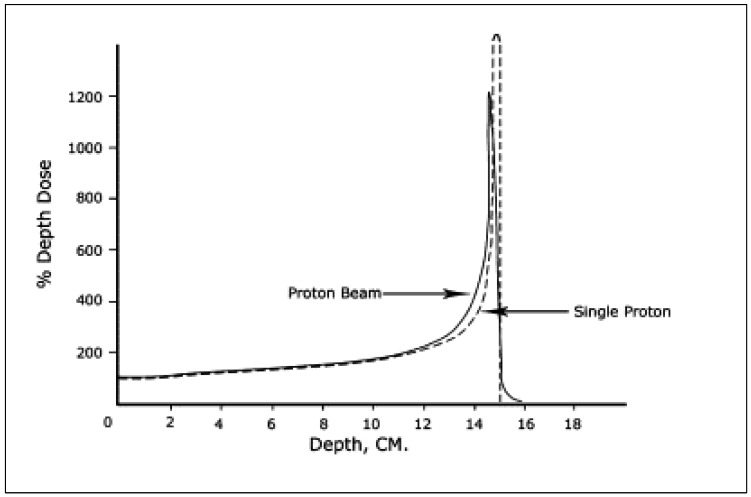
Depth dose curves for a 140 MeV proton beam versus a single proton, highlighting the “Bragg Peak” effect [2].

**Figure 2:**
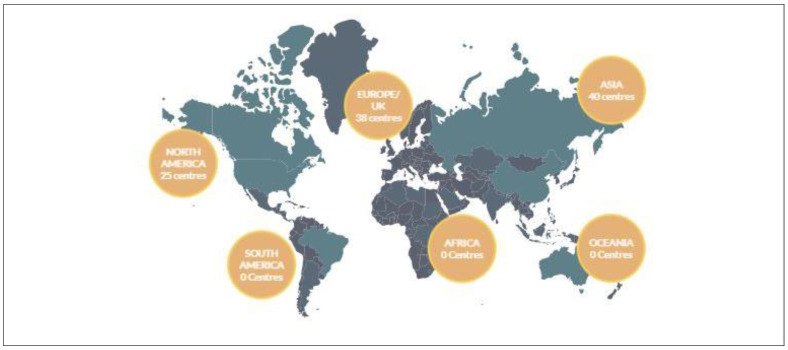
Particle therapy facilities in clinical operation as of October 2022.

Proton therapy holds promise in addressing this limitation, due its unique physical properties. The characteristic behaviour of a proton entails the deposition of the treatment dose at a known depth with a subsequent, rapid dose-fall off. This property, termed the “Bragg peak”, has been utilized to treat a variety of precariously located tumours while sparing low doses to normal surrounding tissue (Figure 1) [2,3]. The enthusiasm surrounding proton therapy stems from this ability to elicit abrupt dose fall-off distal to the target of interest, thereby sparing low doses of radiation to nearby organs at risk. The therapeutic use of protons was first proposed in 1946 by Harvard physicist Robert R. Wilson [3]. The first patients were subsequently treated in 1954 at the Lawrence Berkeley Laboratory in Berkeley, California [4]. The MIT-based Harvard Cyclotron Laboratory began treating patients in 1973 with a focus on uveal melanomas, chordomas and chondrosarcomas involving the skull base and cervical spine [5]. These milestones paved the way for the first hospital-based clinical proton treatment centre, which opened its doors in 1990 at the Loma Linda University Medical Centre [6]. Since that time, the favourable outcomes seen with the use of proton therapy has led to a significant increase in uptake worldwide. According to a recent report from the Particle Therapy Cooperative Group, there are currently 121 proton facilities in operation globally, spanning 21 countries (Figure 2, Table 1). In addition to this, there are another 34 facilities under construction worldwide [7]. This has led to an estimated 280,000 patients that have been treated with proton therapy as of 2021 [7]. However, as we enter this revolutionary new era of radiotherapy, we must ensure that comprehensive clinical expertise, technical fluency and the accompanying biological considerations for particle therapy remain at pace with rapid global implementation.

Breast cancer is the most commonly diagnosed cancer globally with over 2.3 million new cases in 2020. This number is expected to increase by 40% by 2040 [8]. Nearly 65% of patients diagnosed with breast cancer will be treated with radiation [8]. The benefit of adjuvant radiation in patients diagnosed with a breast malignancy has been shown in a number of studies. The Early Breast Cancer Trialists’ Collaborative Group meta-analysis looked at over 10,000 women in 17 clinical trials with the conclusion that radiotherapy after breast conserving surgery results in a 50% reduction in local recurrence and reduces the breast cancer death rate by about one sixth in comparison to women treated with breast conserving surgery alone [9]. Breast radiotherapy for early-stage breast cancer has been well-optimized in recent years to limit normal tissue injury and enhance patient quality-of-life using accelerated partial breast irradiation, deep inspiration breathhold techniques and ultrahypofractionated treatment regimens [10,11,12].

When looking specifically at women with node-positive breast cancers, the benefit of regional nodal irradiation also has been well studied. Level one evidence exists to support the use of regional nodal irradiation in patients with node positive breast cancer to improve locoregional control and disease-free survival [13,14]. However, the increasingly favourable prognosis of this patient population necessitates further care to be taken in mitigating treatment related sequelae. Thus, in the following review, we highlight both clinical and technical considerations for proton radiotherapy delivery in patients with locally advanced breast cancer.

## 2. Current Challenges in Breast Radiotherapy Delivery

The clinical sequalae of radiation treatment for patients with breast cancer has been well established and includes (but is not limited to) cardiac and pulmonary toxicities. In the above-mentioned Early Breast Cancer Trialists collaborative group meta-analysis, the survival benefit of breast radiotherapy was countered by an increased risk of cardiac death [9]. The specific impact of excess radiation dose to the heart was further quantified in a landmark study published by Darby and colleagues which looked at the rates of post-radiotherapy major coronary events, defined as myocardial infarction, coronary revascularization, or death from ischemic heart disease. This analysis showed a 7.4% linear increase in major coronary events with each 1 Gray (Gy) increase in mean heart dose. Notably, the risk was present at all dose levels, with no threshold below which radiation did not impact the heart [15]. The absolute increase in major coronary events was 0.3–0.6% by age 80 depending upon radiation dose exposure, baseline cardiac risk factors, and age at radiation delivery.

More recent studies utilizing three-dimensional imaging have highlighted the relationship between cardiac substructure dose exposure and subsequent cardiac events, with low dose to the left ventricle (V5) most associated with subsequent cardiac events [16]. Further study identified that the mean dose (EQD2) to the left anterior descending artery (LAD) of 2.8 Gy was a threshold for the development of consequent cardiac toxicity. The impact of excessive radiation dose to the lungs has also been established and includes the risk of secondary malignancy as well as radiation pneumonitis. In a population-based, nested case-controlled study of over 20,000 patients treated with radiotherapy for breast cancer, the risk of secondary lung cancers was found to increase linearly with each 8.5 Gy increase in radiation dose [17]. The risk of radiation pneumonitis was reviewed in a meta-analysis that suggested the consideration of alternate treatment modalities in settings where the volume of the ipsilateral lung receiving 20 Gy exceeded 30% or when the mean lung dose was greater than 15 Gy [18].

It has been noted that these dosimetric thresholds are most often breached in the setting of locally advanced breast cancers, given the need to treat nodal regions located adjacent to the cardiac and pulmonary structures [18]. In particular, treatment of the internal mammary nodes can pose a challenge due to the proximity to the heart and LAD. Traditional approaches have used a mix of photons and electrons to address this area, leading to significant dose inhomogeneity. More modern approaches have focused on incorporating tangential intensity modulated radiotherapy (IMRT) and/or volumetric arc therapy (VMAT) to provide better dose distributions. However, the use of IMRT or VMAT, while limiting high doses of radiation to portions of the heart, still leads to an increase in low dose “splash” throughout the thorax including the heart, contralateral breast and lung, the latter of which carries a theoretical increased risk of second malignancy [19]. Thus, despite significant advances in conventional radiotherapy approaches, a need exists to further optimize radiotherapy delivery in patients with locally advanced breast cancer.

## 3. Rationale for Proton Therapy

**Figure 3:**
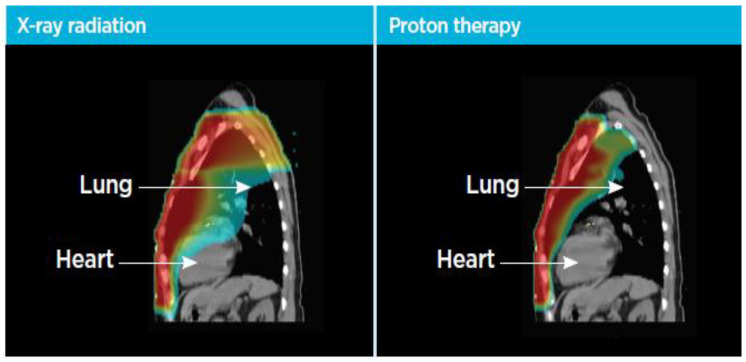
An illustration highlighting differences in cardiac and pulmonary structures with the use of photon radiotherapy versus proton radiotherapy (Figure courtesy of Dr. T.G. Neilan).

**Figure 4:**
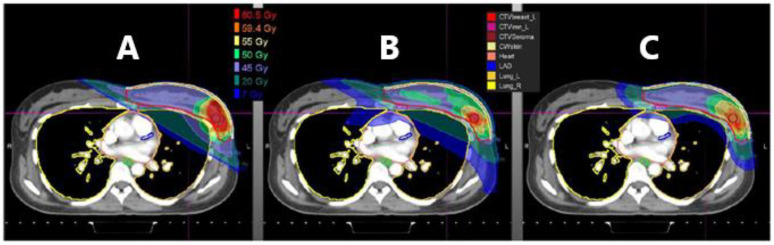
An illustration highlighting differences in the use of (**A**) 3D conformal photon radiotherapy versus (**B**) Volumetric Modulated Arc Therapy (VMAT) versus (**C**) proton radiotherapy (Figure courtesy of Dr. R.B. Jimenez).

Proton therapy holds promise in addressing issues of dose inhomogeneity, in addition to decreasing the dose to both cardiac and pulmonary structures. An illustration highlighting the expected dose distribution between a photon radiotherapy approaches and proton therapy plan is shown in Figure 3 and Figure 4, demonstrating the reduction in lung and heart dose that this modality may provide. Numerous dosimetric comparisons using diverse patients with locally advanced breast cancer requiring regional nodal irradiation have highlighted the cardiopulmonary advantages of proton therapy [20,21,22,23,24,25]. One dose modelling study involving 41 patients treated with comprehensive nodal irradiation modelled dose deposition with both photon and proton planning. Risks were then estimated using the model developed by Darby et al., noted above. This modelling study reported that compared with photon radiotherapy, proton radiation was estimated to decrease the risk of recurrence by 0.9% and lower the risk of acute coronary events by up to 2.9% [20].

While prospective data are limited, early results with proton therapy for locally advanced breast cancer appear promising. For example, in a phase II study of 69 patients undergoing regional nodal irradiation using proton radiotherapy, oncologic outcomes were consistent with photon therapy and importantly, no concerning cardiac changes were identified using strain echocardiography or cardiac biomarkers within 2 months of completion of treatment [21]. This stands in contrast to prior studies of early cardiac changes identified on strain echocardiography and cardiac biomarkers after conventional radiation [22,23,24]. Additional prospective data with direct comparison to conventional radiation, however, are needed [25].

Retrospective dose modelling studies also suggest a benefit of proton therapy in optimizing lung doses with a subsequent decrease in second primary lung cancer risk. One retrospective analysis concluded that the use of proton therapy, using pencil beam scanning techniques, was associated with a lower risk of secondary lung and contralateral breast cancer risk in comparison to both conventional and IMRT/VMAT photon approaches [26]. In the previously mentioned phase II study of 69 patients undergoing regional nodal irradiation using proton radiotherapy, only one patient was found to develop grade 2 radiation pneumonitis. No patients developed grade 3 or 4 radiation pneumonitis [21]. Other reports of proton therapy for breast cancer support this finding [27,28].

The challenges of radiotherapy in the setting of locally advanced breast cancers can be further complicated in the setting of breast reconstruction. The rates of postmastectomy reconstruction for breast cancer patients continues to increase [29]. Radiotherapy delivery in this context involves unique challenges due to the positioning of the prosthesis and subsequent increase in contralateral breast exposure. This issue is compounded in situations where the regional nodal beds require treatment. The use of proton therapy in this setting has been shown to improve dosimetric parameters. One notable clinical study looked at 51 patients treated with intensity modulated proton therapy after breast reconstruction at the Mayo clinic. They found that the use of proton therapy led to improved dosimetric variables and acceptable reconstruction outcomes in comparison to historical data of photon-based approaches [30]. Other data also lend support for these findings [21]. The above studies highlight the promise of proton therapy in decreasing radiotherapy-associated toxicity for women with locally advanced breast cancers, particularly those with breast reconstruction.

## 4. Areas of Uncertainty in Proton Radiation Implementation

As noted above, the primary potential benefit of proton therapy stems from the ability to spare radiation dose to critical organs due to the sharp dose fall-off [31]. Considering this physical property, precise delineation of both targets and organs at risk is required. Care is required when extrapolating current contouring guidelines from photons to protons in the setting of different dose depositions. For example, traditional photon planning of breast regional nodal regions often results in dose deposition to the posterolateral supraclavicular fossa, the low (level I) axilla and the skin, despite these areas not being explicitly contoured in common contouring guidelines. Given that these regions may be potential sites of residual microscopic disease, uncertainty remains regarding the extrapolation of photon contouring guidelines [32]. In addition to accurate target coverage, care must be taken to precisely delineate and avoid organs at risk. For example, breast or chest wall target volumes should not encompass the intercostal muscles or ribs posteriorly in the absence of direct tumour extension to ensure dose fall-off anterior to the heart and lungs. The skin of the chest wall should be separately contoured, and doses carefully considered, acknowledging the lack of skin sparing with proton therapy, to avoid undue acute toxicity. Patients presenting with locally advanced disease may also benefit from the fusion of pre-treatment positron emission tomography imaging to the CT simulation scans to ensure accurate target coverage. Additionally, given the sharp dose fall-off of proton therapy, there is a greater potential impact of interfraction and intrafraction changes [33]. As such, approaches for mitigating these impacts such as image guidance and plan robustness may be required to account for these uncertainties. The evidence surrounding the translation of current photon-related practices into proton radiation delivery is complex and continues to evolve.

One important example of this involves linear energy transfer (LET) with proton therapy. While protons are considered to have a low LET similar to photons and therefore, comparable biologic effects, the use of proton therapy in breast cancer has provided valuable insights regarding the clinical impact of LET variability at the end of range. The relative biological effectiveness (RBE) of protons is defined as the ratio of the dose of a photon reference beam required to produce a specific biological effect to the dose of a proton beam required to produce that same effect. Convention has dictated a generic value of 1.1. However, recent studies suggest that this assumption may be inaccurate. Specifically, data from multiple studies and across multiple centres suggest that the RBE may be closer to 1.2–1.4 at the Bragg peak and distal beam edge where the LET is greatest [34]. The clinical impact of this discrepancy was highlighted in a prospective study of patients treated with proton radiotherapy which reported a rib fracture rate of nearly 7%, which far exceeds rates seen in traditional photon radiation. This rate was even higher, 21%, among patients who received proton therapy for inflammatory carcinoma [27]. Subsequent evaluation of patients treated on the prospective trial was suggestive of an increased RBE at the distal edge of the proton beams accounting for the fracture rate [35]. The incorporation of LET weighted biological dose among women receiving proton therapy for breast cancer in other studies also supported an enhanced biological dose, exceeding 1.1, not only for the ribs, but also to the heart and brachial plexus [36]. This finding is sobering as it suggests that the potential for cardiac sparing as well as other normal tissue sparing with proton therapy could be at least partly mitigated if LET is not appropriately incorporated. Additional study is needed to further optimize our understanding of RBE modelling and optimize the therapeutic ratio for patients [37].

## 5. Ongoing Trials

A number of clinical trials are currently underway to further elucidate the benefits and risks of proton therapy in the setting of locally advanced breast cancer. Amongst these trials are the RTOG 3510 (RADCOMP) Trial [38], the Danish Breast Cancer Group phase 3 randomized breast cancer trial [39] and the UK-based ISRCTN14220944 trial [40]. The primary objective of these randomized clinical trials is to compare the effectiveness of proton beam radiation versus traditional photon therapy in decreasing the risk of major cardiovascular events in patients diagnosed with breast malignancies. Secondary outcomes vary per trial and are focused on several aspects of quality of life, disease control and survival. Eligibility criteria are compared in Table 2, showing the different potential information that each trial will provide. In addition to these large-scale randomized studies, additional endeavours are being undertaken to assess specific aspects of treatment delivery, such as the use of hypofractionation [41] and specific patient subsets, such as those with breast reconstruction [42]. Collectively, these studies will help to guide future patient selection for proton therapy in the setting of breast cancer.

## 6. Conclusions

Throughout this review, we highlight a subset of the literature to help guide clinician decision making and facilitate evidence-based discussions regarding the provision of proton therapy in the setting of locally advanced breast cancer. Patients with locally advanced breast cancers comprise a growing number of patients globally. This unique subset of our patient population entails an increased complexity of planning due to the need for comprehensive nodal coverage and the higher likelihood of breast reconstruction. The favourable prognosis of this group might benefit from vigilant consideration of modalities that may mitigate treatment related toxicity, including cardiopulmonary sequelae. The burgeoning field of proton radiotherapy in this setting holds promise for improving target coverage and decreasing dose to the organs at risk. The evidence surrounding appropriate target volumes and planning approaches, taking into account the unique physical properties of protons, continues to evolve. Further study is needed to appropriately harness this promising technology in service to our patients.

## Figures and Tables

**Table 1:** Particle therapy facilities in clinical operation as of October 2022.

Region	Number of Facilities in Current Operation
North America	25
Europe/UK	38
Asia	40
South America	0
Africa	0
Oceana	0

**Table 2:** A comparison of eligibility criteria between the Pragmatic Randomized Trial of Proton versus Photon Therapy for Patients With Non-Metastatic Breast Cancer (RADCOMP), The Danish Breast Cancer Group phase 3 Randomized Breast Cancer Trial (DBCG) and the UK ISRCTN14220944 trial.

INCLUSION CRITERIA	RADCOMP	DBCG	UK
Age range	≥21 years old	≥18 years old	≥18 years old
Gender	Females and males	Females and males	Females and males
Histology	Invasive mammary carcinoma	Invasive breast cancer or DCIS	Histologically proven invasive breast carcinoma
Stage	Clinical or pathological stage I-III; stage yp 0-III; loco-regional recurrence; Non-metastatic (AJCC 7th)	pTis-4, pN0-N3, M0	
Surgical treatment	Post lumpectomy or any type of mastectomy; any axillary surgery; with or without any type of reconstruction	Post lumpectomy or any type of mastectomy; any axillary surgery; with or without any type of reconstruction (except metal implants)	wide local excision or mastectomy, and any type of axillary surgery
Laterality	Right, left, and bilateral	Right, left, and bilateral	
Radiotherapy	Undergoes adjuvant loco-regional radiotherapy including IMN	Undergoes adjuvant loco-regional radiotherapy including IMN with a plan to fulfill a V95% ≥ 95% of CTVp_breast/chest wall, and if nodal radiotherapy is indicated V90% ≥ 95% of CTV_IMN and V90 ≥ 95% of CTVn Patient is candidate for Dmean heart of ≥4 Gy with photon radiotherapy and/or a V20 ipsilateral lung of ≥37% For patients <41 years old, the medial quadrants of the contralateral breast should be kept <1 Gy Breast, chest wall, or nodal boost is permitted	Recommended to undergo RT to the breast/chest wall + IMN RT; or if pectus excavatum, recommended to undergo RT to the breast/chest wall +/− IMN RT
Days since last history and physical examination	Within 90 days prior to enrollment	Not specified	
ECOG performance status	0–2 within 90 days prior to enrollment	Not specified	
Health coverage	Any but not through this trial	Not specified	
Others	HIV +ve patients are conditionally eligible Patient’s competence Patient to provide study-specific informal consent prior to study entry	Connective tissue disease, post-operative surgical complications, any breast size and seromas are permitted Adjuvant systemic therapy is according to DBCG guidelines Patient with 5 years or more remission from previous non-breast malignancy and low-risk recurrence can participate Life expectancy minimum 10 years	Estimated lifetime risk of radiation-induced late cardiac toxicity ≥2%
EXCLUSION CRITERIA	Prior radiotherapy to the ipsilateral breast/chestwall/thorax Definitive clinical or radiographic evidence of metastatic disease Dermatomyositis with abnormally elevated CPK level or with an active skin rash or scleroderma Non-malignant diseases that would intervene with patient’s radiotherapy or follow up	Previous breast cancer or DCIS of the breast Metachronous bilateral breast cancer Previous radiotherapy to the chest region Pregnancy or lactation Patients with pacemaker Conditions indicating that the patient cannot undergo radiotherapy Unknown non tissues implants upstream of the target volume Metal implants in the radiation area	

IMN = internal mammary nodes.
